# Invasive *Fascioloides magna* infections impact gut microbiota in a definitive host in Europe

**DOI:** 10.1016/j.ijppaw.2024.101024

**Published:** 2024-11-25

**Authors:** Ramona Fleischer, Marc Velling, Wibke Peters, Tomáš Peterka, Frederik Franke, Pavla Jůnková Vymyslická, Steffen Rehbein, Marco Heurich, Simone Sommer

**Affiliations:** a Institute of Evolutionary Ecology and Conservation Genomics, University of Ulm, Germany; b Faculty of Environment and Natural Resources, University of Freiburg, Germany; c Bavarian State Institute of Forestry, Research Unit Wildlife Biology and Management, Freising, Germany; d Faculty of Forestry and Wood Sciences, Czech University of Life Sciences Prague, Praha - Suchdol, Czech Republic; e Šumava National Park, Vimperk, Czech Republic; f Faculty of Environmental Sciences, Czech University of Life Sciences Prague, Praha – Suchdol, Czech Republic; g Boehringer Ingelheim Vetmedica GmbH, Rohrdorf, Germany; h Department of National Park Monitoring and Animal Management, Bavarian Forest National Park, Germany; i Institute for Forest and Wildlife Management, Inland Norway University of Applied Sciences, Koppang, Norway

**Keywords:** Gut microbiota, Invasive parasite, Wildlife health monitoring, Liver fluke *Fascioloides magna*, Bohemian forest ecosystem

## Abstract

Invasive parasites that expand their natural range can be a threat to wildlife biodiversity and may pose a health risk to non-adapted, naive host species. The invasive giant liver fluke, *Fascioloides magna*, native to North America, has extended its range in Europe and uses mainly red deer (*Cervus elaphus*) as definitive hosts. The penetration of the intestinal barrier by the young flukes to reach the liver via the abdominal cavity as well as the release of fluke metabolism products and excreta with the bile and/or changes in the microbial community of the biliary system may enable the translocation of intestinal bacteria across the intestinal barrier and, in turn, could be associated with inflammation and changes in the intestinal bacterial community. The gut commensal community plays a key role in host nutrition and interacts with cells of the immune system to maintain host health. For this study, the gut bacterial community of red deer infected with *F. magna* and of non-infected red deer from one of the largest forest ecosystems in Central Europe, located on the border between the Czech Republic and Germany, was investigated. The individual fluke burden was associated with changes in the gut microbial composition of the gut of infected individuals, whereas the diversity and composition of the gut bacteria were only slightly different between fluke-infected and uninfected deer. Several bacterial taxa at the genus level were unique to individuals carrying either one or many liver flukes. Our results suggest that the microbiota of red deer is stable to perturbation by low numbers of *F. magna*. However, a larger parasite burden may cause changes in the gut microbial composition in definitive hosts implying that non-invasive fecal microbiome assessments could serve as indicator for wildlife health monitoring.

## Highlights

• Liver fluke infections might affect host gut microbiomes, which are essential for host health.
• Giant liver flukes were detected in 14.83% of 418 red deer hunted in the Bohemian Forest ecosystem.
• Infection intensity shifted beta diversity and affected rare and common bacterial taxa.
• Several bacterial genera were unique to individuals carrying either one or many flukes.
• Non-invasive faecal microbiome evaluation could serve as a health indicator in red deer.

## Graphical abstract

**Figure:**
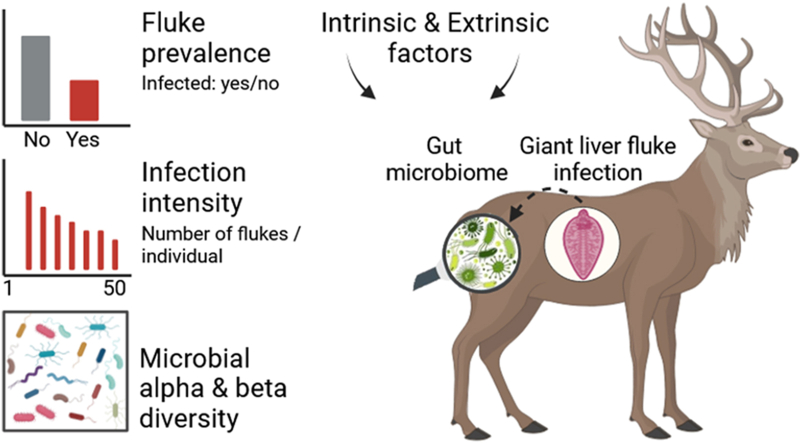

## 1 Introduction

Emerging infectious diseases caused by invasive species threaten wildlife health and conservation (Daszak et al., 2000). Parasites might change host behavior, reduce fitness or even increase mortality (Ezenwa et al., 2015). Parasite infections and associated diseases are frequently linked to a detrimental shift in the gut microbial composition (Carding et al., 2015; Wasimuddin et al., 2018; Levy et al., 2017). The animal gut is colonised by a wide variety of microbes that perform many beneficial functions essential to host health, including energy and fat metabolism, as well as interactions with the host's immune system (Barko et al., 2018; Levy et al., 2017; Lin and Zhang, 2017; Schluter et al., 2020). In healthy individuals commensal gut microbes aid in balancing homeostasis and protect from pathogenic infection (Guzman-Bautista et al., 2020; Lin and Zhang, 2017; Spragge et al., 2023). Shifts in this symbionts' diversity pattern beyond the normal range of variation are maladaptive and often associated with a decrease in beneficial functions, an increase in co-infections, and cause a decline in fitness (Alberdi et al., 2016; Wilkins et al., 2019). Such changes in the gut microbiota might even be present despite the lack of other symptoms of infection in phenotypically healthy individuals (e.g. in bats: Wasimuddin et al., 2018; lemurs: Wasimuddin et al., 2019). As a result, monitoring wildlife gut microbial communities has been identified as a useful marker of host health in wildlife conservation and management (Fackelmann et al., 2023; Risely et al., 2023; Trevelline et al., 2019; West et al., 2019).

In Europe, an expanding invasive parasite is the giant liver fluke (*Fascioloides magna*; Trematoda: Fasciolidae), a parasite that is native to North America, but was introduced, already multiple times, to Europe with infected wapiti (*Cervus elaphus* canadensis) and white-tailed deer (Odocoileus virginianus) (Juhász and Stothard, 2023; Králová-Hromadová et al., 2011). The first European occurrence documented by Bassi (1875) is from the Royal Park La Mandria near Turin in northern Italy. The first occurrence in the Central European foci was reported from the Czech Republic (Ullrich, 1930). Red deer (*Cervus elaphus*) and fallow deer (*Dama*) are the main definitive hosts of *F. magna* in Europe, where the flukes can complete their life cycle and eggs are shed in faeces, but the host is not seriously affected by infection. *F. magna* is among the largest parasites infecting the hepatobiliary system in mammals, and has a complex life cycle. The eggs of giant liver flukes are excreted with the faeces of the final host, embryonate and develop into a ciliated miracidium. In their intermediate hosts, freshwater snails of the Lymnaeidae family, the miracidium then develops through the sporocyst and redia stages into cercariae which are released into the environment, and encyst to become metacercariae. The metacercariae are inadvertently ingested by the final mammalian hosts during feeding. Following excystation in the small intestine of the final host, the newly excysted juvenile flukes penetrate the intestinal wall and migrate to the liver, forming pairs and developing into mature flukes within fibrous capsules and producing eggs (Králová-Hromadová et al., 2016).

In final hosts, the infection may cause gut microbial disturbances related to the penetration of the intestinal barrier, the release of fluke metabolism end products with the bile and/or changes in the microbial community of the biliary system (Pakharukova et al., 2023; Plieskatt et al., 2013; Saltykova et al., 2016). These processes might also enable gut microbes to translocate across the intestinal barrier, potentially causing inflammation, changes in the bacterial composition, and diseases (Vujkovic-Cvijin et al., 2022). Due to the reciprocal relationship between gut microbes and host immunity, shifts in microbial composition may further alter pathogen susceptibility or facilitate co-infections (Clerc et al., 2019; Hoarau et al., 2020; Round and Mazmanian, 2009; Schluter et al., 2020; Zeng et al., 2016). Fluke-infected red deer generally do not show clinical signs or mortality, but spread the parasite in the environment (Juhász and Stothard, 2023), thereby increasing the risk for aberrant hosts, such as several species of livestock (Pybus, 2001; Boggs et al., 2018). In addition, a high parasite load can lead to significant damage to the organs. In fact, changes in the metabolism of fatty acids, oxidative stress, fibrosis, and signalling pathways were linked to *F. magna* infection in red deer (Šimonji et al., 2022). Yet whether *F. magna* infection causes changes in the gut microbiota in one of the main definitive hosts in Europe, the red deer, is unknown.

In this study, we investigated whether *F. magna* infections alter the gut microbiota of red deer sampled in one of the largest forest ecosystems in Central Europe, located at the Czech-German border, including two protected forest areas (Šumava National Park, Bavarian Forest National Park) and one commercially used forest area (Neureichenau State Forest). As red deer are definitive hosts for giant liver flukes and usually do not experience morbidity or mortality, we hypothesise a moderate effect of fluke infection on gut microbial structure, while higher levels of infection may be associated with more pronounced changes in gut microbial communities. This would imply that non-invasive fecal microbiome assessments could serve as an indicator for health monitoring of this large wild ungulate.

## 2 Material and methods

### 2.1 Study species and sample collection

**Fig. 1:**
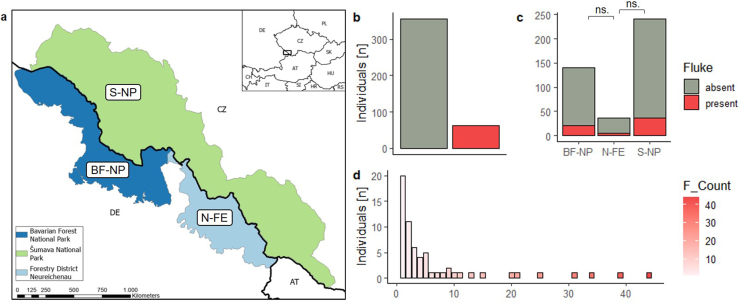
Geographical location of the study area. (a) Map of the three areas (BF-NP=Bavarian Forest National Park, Bavaria, Germany; N-FE=Forest Operation Neureichenau, Bavaria, Germany; S-NP = Šumava National Park, Czech Republic). Giant liver fluke prevalence across (b) all individuals (N = 418) and (c) areas, as well as (d) fluke infection intensity (measured as the individual number of flukes = F_Count) across fluke-infected individuals.

The samples were collected as part of one hunting season from August 2021 to January 2022 in the Bohemian Forest Ecosystem located at the Czech-German border (Heurich et al., 2015), including three areas: the Šumava National Park (S-NP) located in the Czech Republic, the Bavarian Forest National Park (BF-NP) and Neureichenau, a forest enterprise owned by the state of Bavaria (N-FE), both located in Germany (Fig. 1). The vegetation in the forest area consists mainly of coniferous forests (60%), mixed forests (20%), and grasslands including pastures (14%), and to a smaller extent of broadleaved forests (6%), and shrublands (<1%). The main tree species in the study areas include Norway spruce, European beech (*Fagus sylvatica)* silver fir (*Abies alb*a), ash (*Sorbus aucuparia)* and sycamore (*Acer pseudoplatanus*) (Krzystek et al., 2020; van der Knaap et al., 2020).

All fecal and liver samples were collected from deer that were harvested to ensure population control. In order to do so individuals were temporarily attracted to certain spots with supplementary food material. Control of the red deer population is restricted in parts of the forest, approximately 75% and 10 % of the BF-NP and S-NP area, respectively. In BF-NP, most of the population control takes place close to (but not within) winter enclosures (80% according to Möst et al., 2015). Large fenced areas operated to mitigate winter browsing pressure in the tree regeneration and migration to the lower elevations (Möst et al., 2015). A part of the deer population is kept in the enclosures from November until April or May depending on the weather conditions (Belotti et al., 2014; Heurich et al., 2015). Contrary to that hunting close to the enclosures in N-FE under regular German and Bavarian hunting guidelines, in S-NP hunting is restricted by legislation (Janík, 2020; Act No. 449/2001 Coll.). The sex and weight of individuals were recorded, while age was categorized into three groups: adults (A), subadults (SA) and calves (C). Fecal samples were collected directly from the rectum of the animals by the hunters, stored in Nucleic Acid Preservation-buffer and frozen at −20 °C until further processing. The liver of each individual was removed, frozen at approximately −20 C^°^ and shipped to a laboratory for examination for *F. magna* at the end of the hunting season.

### 2.2 Examination of livers for *F. magna*

For examination, the livers were thawed and cut into approximately 1-cm thick slices. Flukes present were recovered and lesions characteristic of *F. magna* infection, such as migratory tracks and presence of melanoid pigment were noted. The slices were then placed overnight in a tub with water and next morning rinsed with water and gently squeezed to expel remaining flukes. The number of flukes per individual was counted to quanitfy infection intensity (F_Count).

### 2.3 16S rRNA gene high throughput sequencing of gut microbiota

Samples were extracted and sequenced at the BIOMES NGS GmbH laboratory in Wildau (Germany). DNA was extracted from frozen pellets using the ZymoBIOMICS 96 DNA Kit and amplified using the paired primers 515F/806R (Caporaso et al., 2010; 2011) for amplification of the V4 region of the bacterial 16S ribosomal RNA (16S rRNA) gene. Paired-end sequencing of the amplicons was performed with the MiSeq (Illumina) over 600 cycles.

The paired-end sequencing results were processed in QIIME 2 (Bolyen et al., 2019) using the DADA2 pipeline (Callahan et al., 2016). Prior analysis we excluded amplicon sequence variants (ASVs) that were not assigned to the kingdom bacteria, unassigned at the phylum level or assigned to chloroplasts and mitochondria.

### 2.4 Statistical analyses

We have examined the microbiome in relation to host intrinsic factors, including age, sex and fluke infection, and extrinsic factors, including the area of origin of the red deer and the hunting season (Table 1).

**Table 1:** Number of available red deer samples and associated host intrinsic (age, sex) and extrinsic (area, hunting month) parameters.

	Age			Sex			Area			*Hunting month*					
	**A**	**SA**	**C**	**F**	**M**	**NA**	**BF-NP**	**N-FE**	**S-NP**	**Aug**	**Sep**	**Oct**	**Nov**	**Dec**	**Jan**
Fluke negative	126	73	157	170	186	0	120	31	205	36	51	60	58	66	85
Fluke positive	47	11	4	34	27	1	20	5	37	5	12	9	14	9	13

We tested for differences in fluke infection prevalence between the areas of origin of the red deer (S-NP, BF-NP, and N-FE), host sexes and age groups, and certain months of the hunting season where there is hunting in Germany and the Czech Republic (from August to January) in a generalized linear model (GLM) with binomial error structure. To assess differences between all levels of categorical variables we re-leveled and re-ran the model.

To investigate whether fluke infection alters gut microbial alpha and beta diversity, we measured alpha diversity using three indices that emphasize distinct features of the gut bacterial communities: the observed number of ASVs represents the count of ASVs, the Shannon Index, which takes into account the abundance of ASVs and the Faith's phylogenetic diversity (PD), that also takes into account the phylogeny of ASVs. For alpha diversity analysis, samples were filtered to a minimum of 10,000 reads (Fig. S1). Next, we tested each alpha diversity metric separately in linear (for observed number of ASVs, Shannon) or generalized (for Faith's PD) linear models. We calculated separate models for 1) fluke prevalence with host sex, area, an interaction between age and fluke infection, as well as hunting season and 2) fluke infection intensity with host sex, age, area, and hunting season as predictors. We have also accounted for an effect of sequencing depth.

We calculated beta diversity based on unweighted and weighted UniFrac distances (Lozupone et al., 2011) and visualized the variables that shape deer beta diversity using a constrained analysis of principal coordinates (CAP), a distance-based redundancy analysis, available in the *vegan* package (Oksanen et al., 2019). For beta diversity analyses all samples were rarefied to a minimum sequence depth of 10,000 (Fig. S1) to normalize for sequencing depth (Weiss et al., 2017). As for alpha diversity, we ran separate models for each distance matrix including 1) host sex, area, an interaction between age and fluke infection, as well as hunting season and 2) host sex, age, area, fluke infection intensity and hunting season as predictors. The models were analyzed in ANOVA-like permutation tests with 9999 permutations using the function *anova.cc)* from the *vegan* package (Oksanen et al., 2019). Since the ordination visualizations illustrating the fluke prevalence and fluke infection intensity models were highly similar concerning unweighted and weighted UniFrac, we only show visualizations from the fluke infection intensity model, but report all statistical model results.

Since differences in gut microbiota were expected to occur between individuals with different parasite loads, we explored differences in the microbiome between adult and subadult deer with a very low infection intensity, i.e. 1 fluke, to individuals that carried a moderate to high parasite load of ≥10 flukes using a subset (N = 28) of the 62 fluke-positive individuals. Further we visualized unique and shared genera between the distinct sampling areas and between individuals with different parasite loads in Venn-diagrams using all, and core (50% prevalence across samples) bacterial taxa.

Finally, we tested for differences in the abundance of bacterial taxa on genus level between BF-NP red deer and S-NP red deer and between red deer with different infection intensities using an ANOVA-Like Differential Expression (ALDEx) procedure implemented in the ALDEx package (Fernandes et al., 2013). We did not test for differential abundance between BF-NP or S-NP and N-FE due to uneven sample sizes.

## 3 Results

### 3.1 *Fascioloides magna* prevalence and infection intensity in red deer

Among all red deer individuals with known fluke infection status (n = 418), 14.83% were infected with *F. magna*. Infected individuals carried between 1 and 44 flukes (mean = 6.5). In our dataset, male and female deer were equally often infected (Table S1, Fig. S2). However, there were clear differences between age groups: subadults were less often infected with flukes than adults and subadults and adults were more often infected than calves (Table S1, Fig. S2). Infection intensity among infected individuals did not vary with the sex or age of the red deer (Table S2). Fluke prevalence and infection intensity among infected individuals was similar across areas and over the hunting season (Table S1, Table S2, Fig. S2).

### 3.2 Effect of intrinsic and extrinsic parameters on gut microbial alpha and beta diversity

**Fig. 2:**
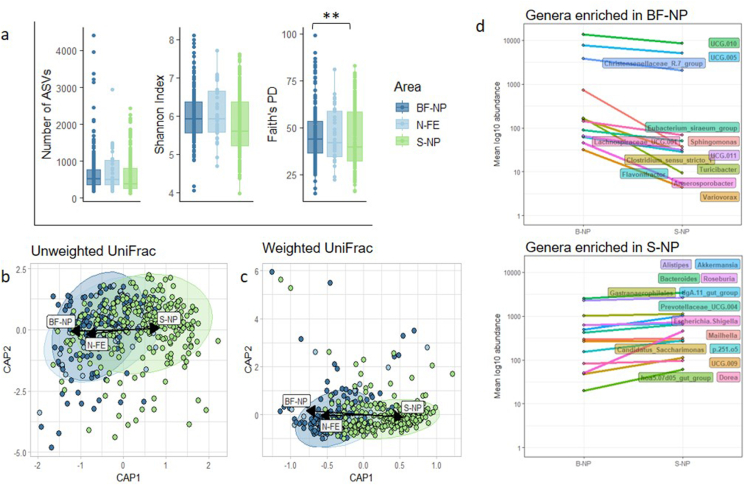
Differences in bacterial diversity and the abundance of specific genera between red deer from the different areas. Gut microbial alpha diversity (a) showed differences in phylogenetic diversity between the areas located in the national parks. Beta diversity depicted differences in (b) rare (unweighted UniFrac) and (c) abundant (weighted UniFrac) bacterial genera across areas. (d) Certain bacterial taxa differed in abundance between BF-NP and S-NP. Each line represents one bacterial genus. (For interpretation of the references to colour in this figure legend, the reader is referred to the Web version of this article.)

Host sex did not shape alpha diversity in terms of the number of ASVs and Shannon Index, but males harboured phylogenetically more diverse gut bacteria than females (Faith's PD). Deer age had no effect. Moreover, alpha diversity in November and December was higher than during the other months (Fig. S3, Table S3). The number of ASVs and Shannon Index did not significantly differ between areas, but deer from the BF-NP carried phylogenetically more diverse gut bacteria than deer from S-NP (Fig. 2a, Table S3).

The beta diversity, i.e. the composition of gut bacteria, was not shaped by host sex, but differed between age groups and over the course of the hunting season (Fig. S3, Table S4). In line with Faith's PD, the composition of rare and abundant gut bacterial taxa varied between the areas (Fig. 2b–Table S4). Specifically, 26 bacterial genera differed in abundance between the red deer of the two National Parks (Fig. 2d). For instance, *Akkermansia, Allstipes* and *Bacteroides* were more abundant in deer from S-NP, and *Sphingomonas, Turibacter* and *Variovarax* were more abundant in the BF-NP deer. Moreover, several bacterial genera were unique to each area (Fig. S4).

### 3.3 Effect of fluke prevalence and infection intensity on the gut microbial community

**Fig. 3:**
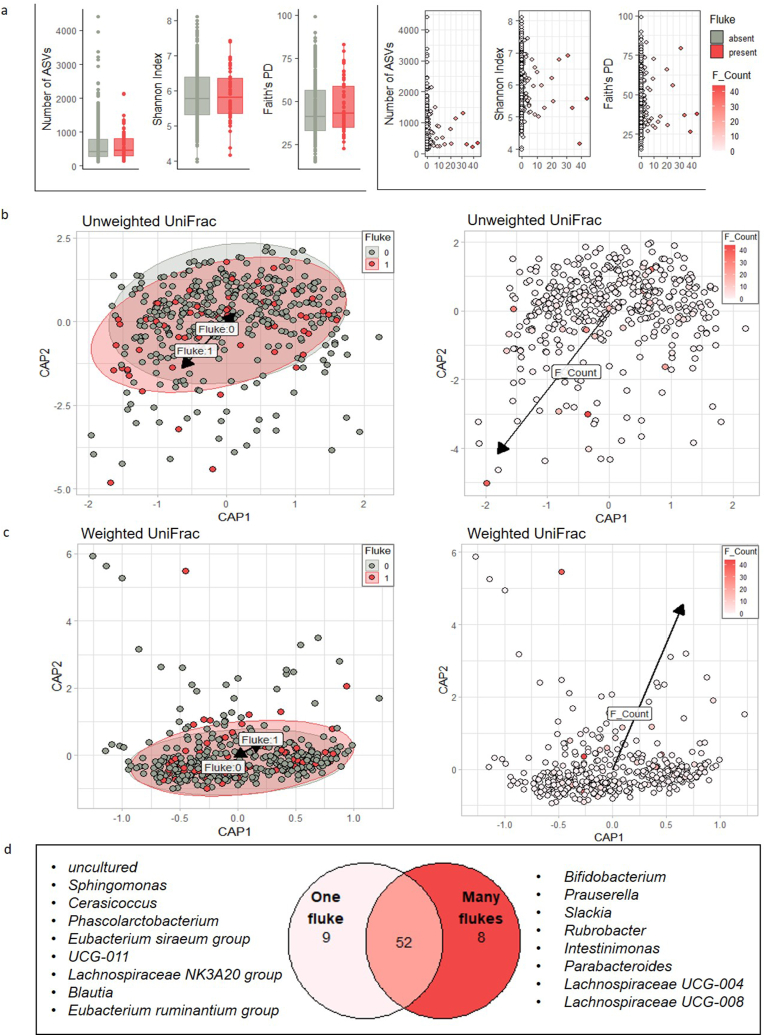
Differences in bacterial diversity in relation to fluke prevalence and infection intensity. Gut microbial alpha diversity (a) according to fluke prevalence and infection intensity. Beta diversity depicting differences in (b) rare (unweighted UniFrac) and (c) abundant (weighted UniFrac) bacterial genera in relation to liver fluke prevalence and infection intensity (left and right panels, respectively). (d) Number of shared and unique core gut bacterial taxa (i.e. taxa with 50% prevalence across individuals) between individuals carrying one or ≥10 flukes at the genus level. Noted are the genus names of the unique genera in each group.

Gut bacterial alpha diversity did not differ between fluke-infected and non-infected individuals in terms of the number of ASVs and Shannon Index, and Faith PD was marginally associated with fluke prevalence (Fig. 3a, Table S3). In terms of beta diversity, rare taxa (unweighted UniFrac), but not common bacterial taxa (weighted UniFrac), differed between uninfected and infected individuals (Fig. 3b and c, Table S4). However, the intensity of fluke infection had a significant effect on both beta diversity metrics (Fig. 3, Table S4). Further comparison of individuals with one fluke with individuals with ≥10 flukes revealed differences in the bacterial composition; the relative abundance of bacterial genera differed at both the individual and group level (Fig. S5). Several bacterial genera were unique to individuals carrying one or ≥10 liver flukes, respectively. These included bacterial taxa with high prevalence across individuals (Fig. 3d–Table S5).

## 4 Discussion

This study aimed to identify whether and how the gut microbiota responds to giant liver fluke infections in red deer, constituting an important definitive host in Europe. Our results demonstrate that while the prevalence of flukes did not influence the alpha diversity of the gut bacteria, and in terms of community composition only rare bacterial taxa were affected, the number of liver flukes carried by an individual was associated with significant alterations in the gut microbiota. These changes affected both rare and common bacterial taxa. In addition, we report profound microbial variation between the two areas in the Bavarian Forest National Park and Šumava National Park.

Giant liver flukes were found in 14.83% of the investigated individuals, with no significant differences across areas, seasons or sexes. The results of a prior investigation in a different region of Germany also indicated that males and females are equally often infected with *F. magna* in multiple deer species (Rehbein et al., 2021). Given that the animals pick up the infection from the environment during feeding or drinking activities, it is reasonable to conclude that with advancing age, exposure to parasites becomes increasingly probable. Although the occurrence of fluke infections in Šumava National Park was documented (Novobilský et al., 2007) prior to their detection in the adjacent areas of the Bavarian Forest National Park in Germany (first detection 2019; Sommer et al., 2022), our findings revealed comparable fluke prevalence across all study areas. This may be attributable to discrepancies in the distribution and prevalence of the intermediate hosts of *F. magna*, lymnaeid snails that are essential for the successful transmission of the fluke between the German and Czech forest areas. This could either facilitate the expansion of the fluke in Germany or impede further expansion in the Czech Republic.

While enteric parasite infections are commonly associated with alterations in gut microbial composition (Wasimuddin et al., 2018), our findings did not indicate a significant impact of the infection with the liver fluke *F. magna* on microbial alpha diversity. This suggests that *F. magna* and red deer have a well-adapted parasite-host relationship. This finding is not unexpected given the close phylogenetic relationship between the European red deer and the natural definitive host, the wapiti (Mackiewicz et al., 2022; Randi et al., 2001). One potential mechanism of adaptation may be mediated via the gut microbiome, as evidenced by the evolution of reduced *Staphylococcus aureus* virulence in response to co-evolution with *Enterococcus faecalis* in a nematode host (Ford et al., 2016). Specifically, *S. aureus* adapts to the microbe by producing fewer iron-scavenging siderophores, which also reduces pathogen virulence and consequently depicts host-protective virulence evolution triggered by certain microbes. In addition, complex microbe-microbe interactions establish and maintain gut homeostasis, which confers a certain level of resistance to perturbation, such as infection with parasites or pathogenic bacteria (Kamada et al., 2013; Khan et al., 2021; Levy et al., 2017; Shi et al., 2019). It is noteworthy that pathogens can also exploit gut microbes and their metabolites to successfully infect the host (e.g., reviewed in Stevens et al., 2021). Although giant liver flukes may not cause significant health issues for red deer, the longevity and high mobility of the host species (Peters et al., 2019; Rivrud et al., 2016) can play a pivotal role in the perpetuation and distribution of the fluke. Indeed, the primary means of dispersing *F. magna* to novel habitats is through natural movement and/or human-mediated translocation of infected red deer (Juhász and Stothard, 2023). Subsequently, this may increase the risk for aberrant hosts, such as sheep (Králová-Hromadová et al., 2016). In livestock, enzootic helminth infections have been estimated to cause costs that are similar to, or higher than, those associated with epizootic diseases (Charlier et al., 2020).

It is possible that parameters other than the presence of an infection may alter the host gut microbiota. The variation in infection severity and duration, as well as the presence of co-infections, may be more significant predictors of pathogen-microbiota associations than the mere presence of a single infection (Schmid et al., 2022). This is exemplified by the case of African buffalo (*Syncerus caffer*), where helminth-tuberculosis co-infection and the duration of tuberculosis infection were found to account for a greater degree of variation in the gut microbiota than the presence of tuberculosis alone (Sabey et al., 2020). In a similar vein, our findings revealed a correlation between the composition of the gut bacterial community and the intensity of fluke infection, defined as the number of flukes carried by an individual. In light of the life cycle of *F. magna* (flukes typically mature in pairs), this suggests that higher fluke loads that penetrate the intestine may facilitate the translocation of gut microbes across the intestinal barrier, potentially leading to disease (Chopyk and Grakoui, 2020) or systemic immune responses. For example, certain gut bacteria are targeted by immunoglobulin G antibodies in human serum (Vujkovic-Cvijin et al., 2022). Furthermore, elevated fluke loads result in an increased prevalence of adult flukes, accompanied by enhanced egg production and the excretion of fluke metabolic end products into the bile. These are subsequently disseminated with the host's faeces (Králová-Hromadová et al., 2016). The data revealed the presence of several bacterial genera that were exclusive to either deer infected with a single fluke or with a minimum of 10 flukes, respectively. This finding suggests that, rather than variation in bacterial abundance, higher infection intensity may result in subtle shifts in the presence and absence of colonising bacteria. This effect may be even more pronounced in highly infected individuals, which were not identified in this dataset. It has been demonstrated that specific bacterial strains can exert a disproportionate influence on the composition of the gut microbiome, thereby affecting its stability and overall health (Banerjee et al., 2018). Additionally, research suggests that the identity of these keystone bacterial strains can vary significantly depending on the host environment and dietary factors (Weiss et al., 2023). Consequently, the loss of specific bacterial genera in the gut environment may create opportunities for the establishment of other bacterial species that could potentially have adverse effects, due to alterations in the microbe-microbe interaction networks.

In addition to the effects of infection intensity, there was substantial variation in the gut microbial composition of red deer among areas, particularly between the two National Parks, which differed in the abundance of 26 bacterial genera. This finding is somewhat unexpected at first glance, given the close geographic proximity and shared location within the Bohemian Forest Ecosystem of both areas. However, in consideration of the landscape structure, it can be observed that BF-NP and S-NP exhibit notable differences in forest cover and types, which may potentially influence the deer's diet (Janík and Romportl, 2017; Krojerova-Prokesova et al., 2010). This, in turn, may shape the composition of the gut microbiota. For example, the composition of the gut microbiota was found to vary depending on habitat characteristics, such as vegetation structure, in Siberian roe deer (*Capreolus pygargus*) in China (Yuan et al., 2023). Furthermore, the bait used to attract deer for population control purposes (this study) may differ between National Parks. The host diet exerts a profound influence on the gut microbiota in vertebrates (Groussin et al., 2017; Kartzinel et al., 2019; Youngblut et al., 2019). Alterations in diet give rise to notable variations in the gut microbial composition observed among distinct deer species (Wang et al., 2023). A previous study among red deer in the BF-NP demonstrated that the gut microbiota differed between free-ranging deer and those in winter enclosures, where supplementary food was provided (Menke et al., 2019). A total of 26 bacterial genera exhibited differential abundance between the National Parks, with 12 displaying greater prevalence in deer from BF-NP and the remaining 14 in deer from S-NP. A considerable number of bacterial taxa identified in wildlife have not been extensively characterized, which impedes the formulation of definitive functional inferences. However, some of the identified bacterial taxa were repeatedly linked to either beneficial functions or disease. For example, Akkermansia, which were more abundant in S-NP, have been linked to beneficial functions such as improved gut barrier functionality. However, they are reduced in humans with obesity, inflammatory bowel disease and other metabolic syndromes (Dao et al., 2016). Red deer from BF-NP exhibited a higher prevalence of Sphingomonas, a potential probiotic genus that has been demonstrated to antagonise Vibrio anguillarum infection in young Labeo rohita (Chaudhary and Qazi, 2014). However, it is important to note that the genus also encompasses pathogens (Ryan and Adley, 2010). The abundance of Sphingomonas was found to be reduced in sheep that were fed a pelleted high-grain diet. This is presumably due to the fact that Sphingomonas is linked to cellulose degradation (Zhang et al., 2021). Thus, in addition to gut parasites and differences in parasite pressure, small changes in diet may also contribute to gut microbial variation among deer from the same forest ecosystem, depending on different management practices.

## 5 Conclusions

The study revealed that an altered composition of gut microbes is linked to the intensity of infection by the giant liver fluke (*F. magna*) in red deer, an important definitive host for this parasite in Europe. This finding may have implications for long-term health outcomes and the potential for co-infections. It is important to note that parasite infections can have significant effects on host health, which may not be fully captured by single-point measurements. Consequently, long-term microbial monitoring in conjunction with the analysis of stress markers and pathogen screening represents a promising avenue of research for elucidating the potential fitness consequences of giant liver fluke infections in definitive hosts that appear to tolerate a high parasite burden. In light of the potential for further *F. magna* spread by red deer and the possibility of shifts in the distribution of intermediate host snails (Juhász and Stothard, 2023), our study provides a baseline for giant liver fluke infections in definitive hosts in a large protected area in central Europe, situated within a human-altered landscape. The monitoring of fecal microbiome composition may prove to be an effective indicator of health in wildlife populations.

## CRediT authorship contribution statement

**Ramona Fleischer:** Writing – review & editing, Writing – original draft, Visualization, Formal analysis. **Marc Velling:** Writing – review & editing, Data curation. **Wibke Peters:** Writing – review & editing, Data curation. **Tomáš Peterka:** Writing – review & editing, Data curation. **Frederik Franke:** Writing – review & editing, Data curation. **Pavla Jůnková Vymyslická:** Writing – review & editing, Data curation. **Steffen Rehbein:** Writing – review & editing, Methodology. **Marco Heurich:** Writing – review & editing, Supervision, Funding acquisition. **Simone Sommer:** Writing – review & editing, Supervision.

## Data and code availability

The data and code for this manuscript are openly available and downloadable at GitHub (https://github.com/rfleischer93/Invasive-liver-fluke-infections-and-gut-microbiota-in-red-deer). The raw 16S sequences will be uploaded to NCBI.

## Funding

This work was financed by the program Ziel ETZ FreeState of Bavaria – Czech Republic 2014–2020 (INTERREG V) (project number 315).

## Conflict of interests

The authors declare no conflicting interests in regard to this paper.
