# Supplementary material for: Invasive Fascioloides magna infections impact gut microbiota in a definitive host in Europe
Source: Int J Parasitol Parasites Wildl. 2024 Nov 25;25:101024. doi: 10.1016/j.ijppaw.2024.101024 (PMC11648883; doi:10.1016/j.ijppaw.2024.101024)
Supplement: Multimedia component 1 [file mmc1.docx]

Supplementary Material

# Invasive *Fascioloides magna* infections impact gut microbiota in a definitive host in Europe

Ramona Fleischer^1*^, Marc Velling^2,8^, Wibke Peters^3^, Tomáš Peterka^4,5^, Frederik Franke^3^, Pavla Jůnková Vymyslická^5,6^, Steffen Rehbein^7^, Marco Heurich^2,8,9^, Simone Sommer^1^

^1^ Institute of Evolutionary Ecology and Conservation Genomics, University of Ulm, Germany

^2^ Faculty of Environment and Natural Resources, University of Freiburg, Germany

^3^ Bavarian State Institute of Forestry, Research Unit Wildlife Biology and Management, Freising, Germany

^4^ Faculty of Forestry and Wood Sciences, Czech University of Life Sciences Prague, Praha - Suchdol, Czech Republic

^5^ Šumava National Park, Vimperk, Czech Republic

^6^ Faculty of Environmental Sciences, Czech University of Life Sciences Prague, Praha – Suchdol, Czech Republic

^7^ Boehringer Ingelheim Vetmedica GmbH, Rohrdorf, Germany

^8^ Department of National Park Monitoring and Animal Management, Bavarian Forest National Park, Germany

^9^ Institute for Forest and Wildlife Management, Inland Norway University of Applied Sciences, Koppang, Norway

* corresponding author: ramona.fleischer@uni-ulm.de


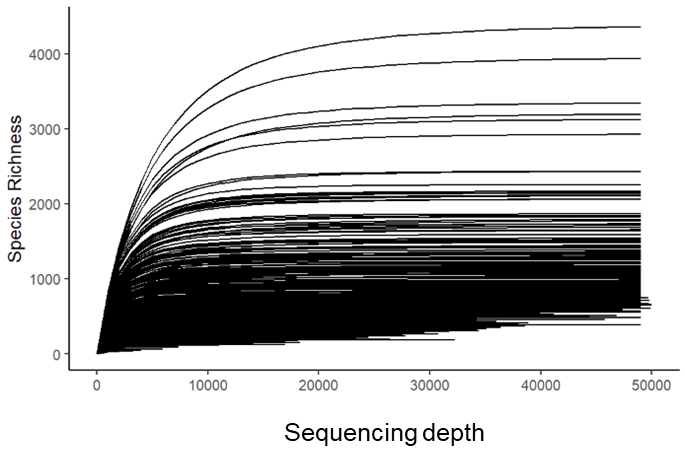


**Fig. S1** Rarefaction curve showing the number of microbial ASVs detected with increasing number of reads in each red deer sample.


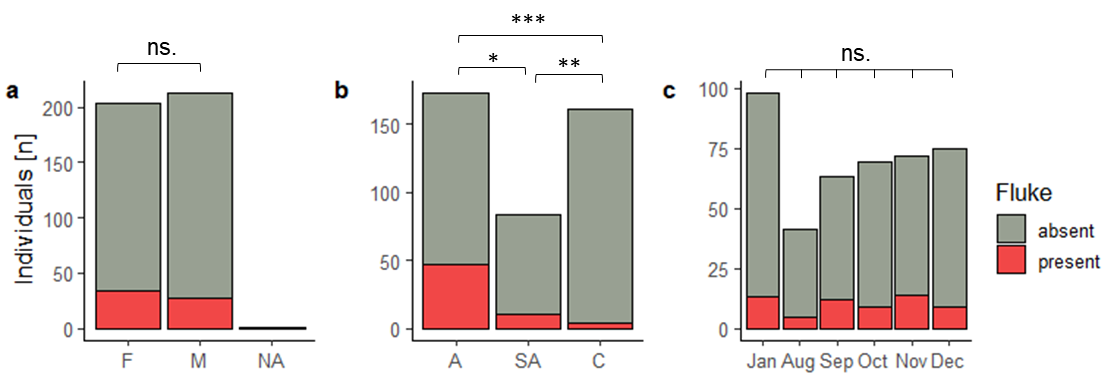


**Fig. S2** Liver fluke prevalence in red deer in relation to host sex, age and hunting season tested with a generalized linear model. Significance codes: *** p < 0.001, ** p < 0.01, * p < 0.05. Host age groups include adults= A, subadults=SA and calves=C. Sampling areas include the National Park Šumava= S-NP, the Bavarian Forest National Park= BF-NP and a forest enterprise owned by the state of Bavaria= N-FE. The hunting season ranged from August-January.

**
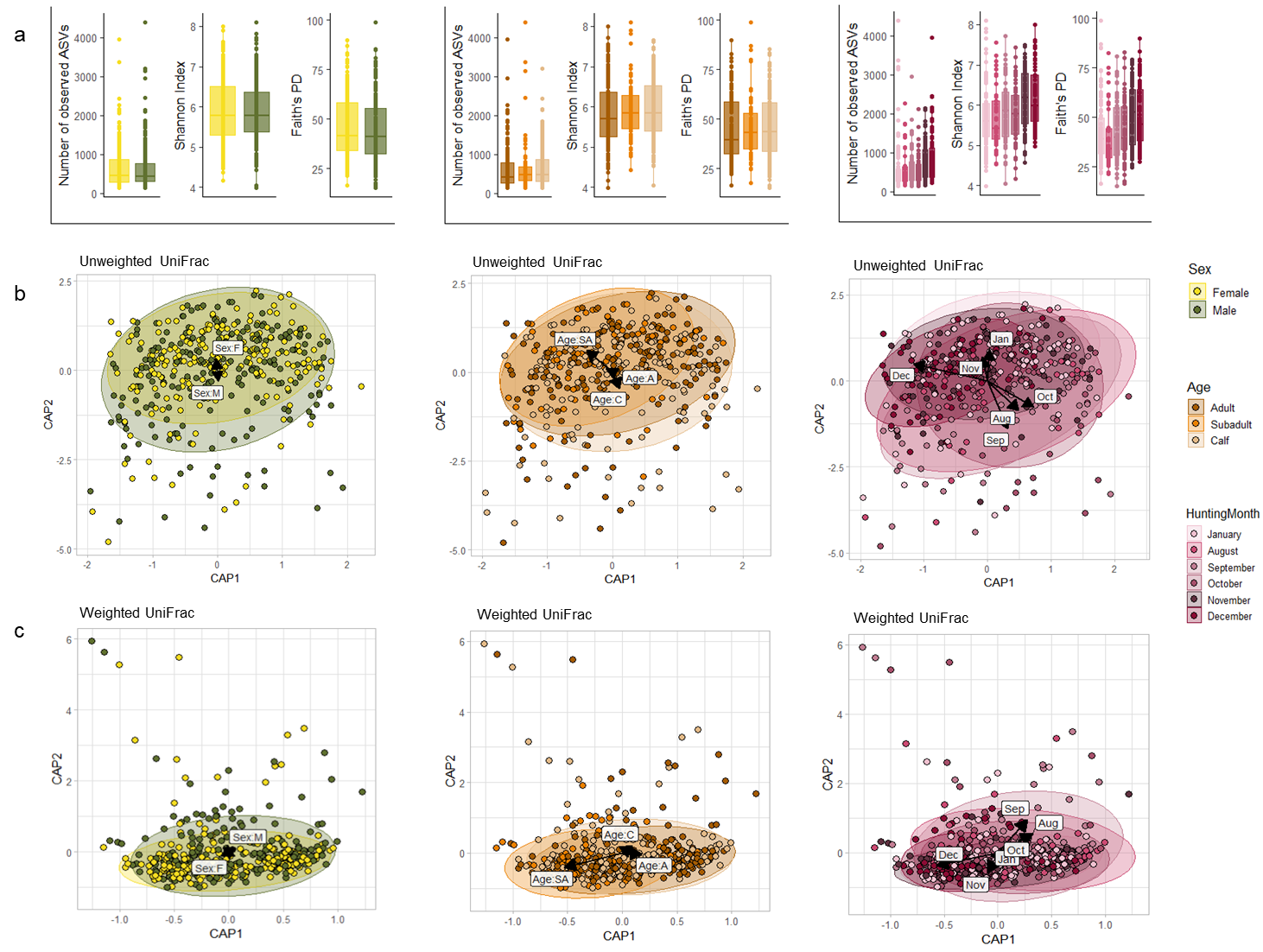

Fig. S3** Gut bacterial alpha and beta diversity according to host intrinsic and extrinsic factors. Displayed are the alpha diversity metrices a) Number of observed ASVs, Shannon, and Faith PD, and beta diversity metrices based b) on unweighted UniFrac, and c) on weighted UniFrac distances in relation to sex, age and hunting month (from left to right).


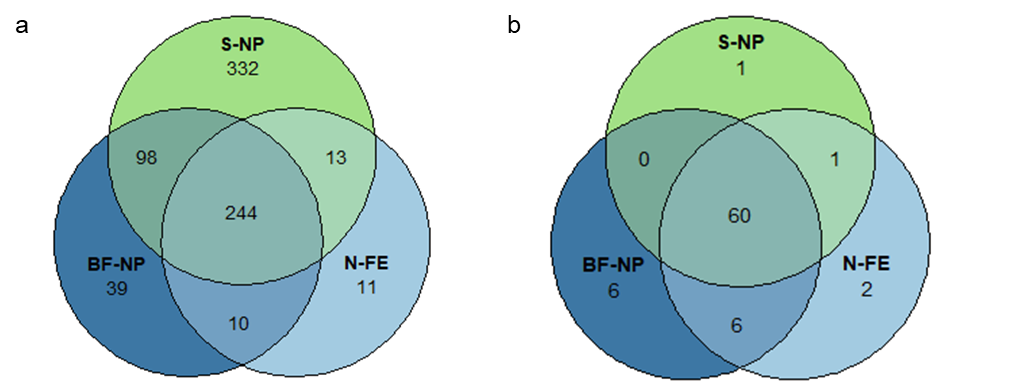


**Fig. S4** Venn diagrams indicating unique and shared bacterial taxa (on genus level) in the gut of red deer hunted in distinct areas of the Bohemian National Park: BF-NP located in the Bavarian Forest National Park, Germany; S-NP located in the Šumava National Park, Czech Republic; and a forest enterprise located in a commercial state forest in Bavaria, Germany (N-FE). Venn diagrams are displayed across a) all bacterial taxa on genus level, and b) core genera with at least 50% prevalence.


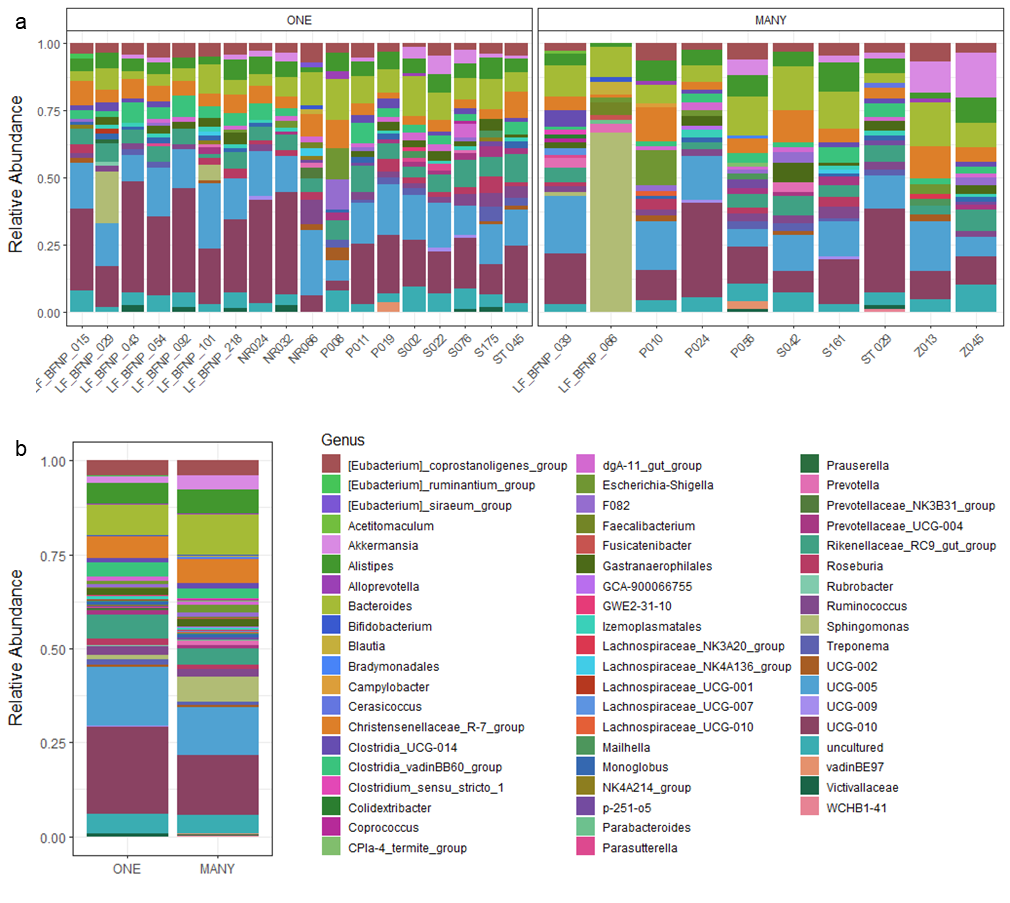


**Fig. S5** Relative abundance of bacterial genera in individuals with one liver fluke and with many (≥10) liver flukes. Composite bar charts a) on individual level and b) on group level. Displayed are only genera with a minimum abundance of 1%.

**Table S1** Effect of intrinsic (host sex, age) and extrinsic factors (area, hunting season) on fluke prevalence in red deer, calculated with generalized linear models using binomial error distribution. Significant p-values in bold. Significance codes: *** p < 0.001, ** p < 0.01, * p < 0.05. Host age groups include adults= A, subadults=SA and calves=C. Sampling areas include the National Park Šumava= S-NP, the Bavarian Forest National Park= BF-NP and a forest enterprise owned by the state of Bavaria= N-FE. The hunting season ranged from August-January.

|  | Estimate | Std. Error | p-value |
| --- | --- | --- | --- |
| Sex:M | 0.05 | 0.34 | 0.888 |
| Area:N-FE / BF-NP | -0.85 | 0.64 | 0.182 |
| Area:S-NP / BF-NP | -0.27 | 0.37 | 0.472 |
| Area: N-FE / S-NP | -0.59 | -0.98 | 0.328 |
| Age:SA / A | -1.02 | 0.46 | **0.027 *** |
| Age:C / A | -2.74 | 0.55 | **5.15e-07 ***** |
| Age:C / SA | -1.72 | 0.63 | **0.006 **** |
| Season:Aug / Jan | -0.11 | 0.60 | 0.852 |
| Season:Sep / Jan | 0.62 | 0.50 | 0.213 |
| Season:Oct / Jan | 0.27 | 0.50 | 0.594 |
| Season:Nov / Jan | 0.71 | 0.46 | 0.122 |
| Season:Dec / Jan | 0.09 | 0.50 | 0.857 |
| Season:Sep / Aug | 0.73 | 0.63 | 0.245 |
| Season:Oct / Aug | 0.38 | 0.63 | 0.549 |
| Season:Nov / Aug | 0.82 | 0.60 | 0.171 |
| Season:Dec / Aug | 0.20 | 0.64 | 0.753 |
| Season:Oct / Sep | -0.35 | 0.52 | 0.504 |
| Season:Nov / Sep | 0.09 | 0.48 | 0.845 |
| Season:Dec / Sep | -0.53 | 0.53 | 0.324 |
| Season:Nov / Oct | 0.44 | 0.89 | 0.373 |
| Season:Dec / Oct | -0.18 | -0.33 | 0.745 |
| Season:Dec / Nov | -0.62 | -1.25 | 0.212 |

**Table S2** Effect of intrinsic (host sex, age) and extrinsic (area and hunting season) factors on fluke infection intensity in red deer, calculated with generalized linear models using Gamma error distribution. Significant p-values in bold. Significance codes: *** p < 0.001, ** p < 0.01, * p < 0.05. Host age groups include adults= A, subadults=SA and calves=C. Sampling areas include the National Park Šumava= S-NP, the Bavarian Forest National Park= BF-NP and a forest enterprise owned by the state of Bavaria= N-FE. The hunting season ranged from August-January.

|  | Estimate | Std. Error | p-value |
| --- | --- | --- | --- |
| Sex:M | -0.04 | 0.06 | 0.547 |
| Area:S-NP / BF-NP | 0.02 | 0.06 | 0.771 |
| Area:N-FE / BF-NP | 0.13 | 0.18 | 0.465 |
| Area:N-FE / S-NP | 0.11 | 0.18 | 0.517 |
| Age:A / SA | -0.23 | 0.14 | 0.104 |
| Age:C / A | -0.01 | 0.07 | 0.923 |
| Age:C / SA | -0.24 | 0.14 | 0.094 |
| Season:Aug / Jan | 0.46 | 0.30 | 0.131 |
| Season:Sep / Jan | 0.02 | 0.07 | 0.819 |
| Season:Oct / Jan | -0.03 | 0.05 | 0.533 |
| Season:Nov / Jan | -0.001 | 0.06 | 0.992 |
| Season:Dec / Jan | 0.28 | 0.18 | 0.119 |
| Season:Sep / Aug | -0.45 | 0.31 | 0.148 |
| Season:Oct / Aug | -0.50 | 0.30 | 0.106 |
| Season:Nov / Aug | -0.46 | 0.30 | 0.130 |
| Season:Dec / Aug | -0.18 | 0.34 | 0.597 |
| Season:Oct / Sep | -0.05 | 0.06 | 0.455 |
| Season:Nov / Sep | -0.02 | 0.08 | 0.840 |
| Season:Dec / Sep | 0.26 | 0.18 | 0.142 |
| Season:Nov / Oct | 0.03 | 0.06 | 0.573 |
| Season:Dec / Oct | 0.31 | 0.17 | 0.080 |
| Season:Dec / Nov | 0.28 | 0.18 | 0.125 |

**Table S3** Effect of intrinsic (host sex, age) and extrinsic factors (area and hunting season) and liver fluke infections on three metrics of microbial alpha diversity in red deer with a) Number of observed ASVs, b) Shannon Index and c) Faiths PD. Significant p-values in bold. Significance codes: *** p < 0.001, ** p < 0.01, * p < 0.05. Host age groups include adults=A, subadults=SA and calves=C. Sampling areas include the National Park Šumava=S-NP, the Bavarian Forest National Park=BF-NP and a forest enterprise owned by the state of Bavaria=N-FE. The hunting season ranged from August-January.

1. Observed ASVs

|  | Estimate | Std. Error | p-value |
| --- | --- | --- | --- |
| Sex:M | -1.58e-02 | 1.57e-02 | 0.315 |
| Area:S-NP / BF-NP | -3.47e-02 | 1.80e-02 | 0.054 |
| Area:N-FE / BF-NP | 3.87e-03 | 2.86e-02 | 0.892 |
| Area:N-FE / S-NP | 3.86e-02 | 2.72e-02 | 0.156 |
| Age:C / SA | 3.54e-02 | 2.26e-02 | 0.118 |
| Age:A / SA | 2.07e-02 | 2.49e-02 | 0.405 |
| Age:A / C | -1.47e-02 | 1.79e-02 | 0.413 |
| Fluke-infected | 7.35e-02 | 4.72e-02 | 0.120 |
| Season:Aug / Jan | -5.57e-03 | 2.73e-02 | 0.838 |
| Season:Sep / Jan | 2.73e-02 | 2.42e-02 | 0.260 |
| Season:Oct / Jan | 3.69e-02 | 2.31e-02 | 0.111 |
| Season:Nov / Jan | 9.38e-02 | 2.30e-02 | **5.38e-05 ***** |
| Season:Dec / Jan | 1.06e-01 | 2.29e-02 | **4.97e-06 ***** |
| Season:Sep / Aug | 3.29e-02 | 2.97e-02 | 0.270 |
| Season:Oct / Aug | 4.25e-02 | 2.89e-02 | 0.143 |
| Season:Nov / Aug | 9.94e-02 | 2.90e-02 | **0.001 ***** |
| Season:Dec / Aug | 1.12e-01 | 2.90e-02 | **0.0001 ***** |
| Season:Oct / Sep | 9.61e-03 | 2.56e-02 | 0.708 |
| Season:Nov / Sep | 6.66e-02 | 2.54e-02 | **0.009 **** |
| Season:Dec / Sep | 7.87e-02 | 2.59e-02 | **0.003 **** |
| Season:Nov / Oct | 5.70e-02 | 2.47e-02 | **0.022 *** |
| Season:Dec / Oct | 6.91e-02 | 2.52e-02 | **0.006 **** |
| Season:Dec / Nov | 1.21e-02 | 2.45e-02 | 0.621 |
| Seqdepth | 5.20e-06 | 1.42e-07 | **< 2e-16 ***** |
| Age:C:Fluke-infected | -1.90e-02 | 8.80e-02 | 0.829 |
| Age:A:Fluke-infected | -4.87e-02 | 5.35e-02 | 0.363 |
| Age:SA: Fluke-infected | 1.90e-02 | 8.80e-02 | 0.829 |

1. Shannon Index

|  | Estimate | Std. Error | p-value |
| --- | --- | --- | --- |
| Sex:M | -3.83e-02 | 4.22e-02 | 0.365 |
| Area:S-NP / BF-NP | -7.94e-02 | 4.83e-02 | 0.101 |
| Area:N-FE / BF-NP | 4.44e-02 | 7.69e-02 | 0.564 |
| Area:N-FE / S-NP | 1.24e-01 | 7.32e-02 | 0.092 |
| Age:C / SA | 8.40e-02 | 6.07e-02 | 0.167 |
| Age:A / SA | 3.75e-02 | 6.70e-02 | 0.576 |
| Age:SA / C | -8.40e-02 | 6.07e-02 | 0.167 |
| Fluke-infected | 1.53e-01 | 1.27e-01 | 0.230 |
| Season:Aug / Jan | -9.01e-03 | 7.34e-02 | 0.902 |
| Season:Sep / Jan | 5.54e-02 | 6.51e-02 | 0.395 |
| Season:Oct / Jan | 8.54e-02 | 6.22e-02 | 0.170 |
| Season:Nov / Jan | 2.31e-01 | 6.19e-02 | **0.0002 ***** |
| Season:Dec / Jan | 2.67e-01 | 6.16e-02 | **1.84e-05 ***** |
| Season:Sep / Aug | 6.44e-02 | 8.01e-02 | 0.421 |
| Season:Oct / Aug | 9.45e-02 | 7.78e-02 | 0.226 |
| Season:Nov / Aug | 2.40e-01 | 7.81e-02 | **0.002 **** |
| Season:Dec / Aug | 2.76e-01 | 7.82e-02 | **0.0005 ***** |
| Season:Oct / Sep | 3.00e-02 | 6.90e-02 | 0.664 |
| Season:Nov / Sep | 1.75e-01 | 6.83e-02 | **0.011 *** |
| Season:Dec / Sep | 2.12e-01 | 6.98e-02 | **0.003 **** |
| Season:Nov / Oct | 1.45e-01 | 6.66e-02 | **0.030 *** |
| Season:Dec / Oct | 1.82e-01 | 6.78e-02 | **0.008 **** |
| Season:Dec / Nov | 3.66e-02 | 6.61e-02 | 0.580 |
| Seqdepth | 1.26e-05 | 3.83e-07 | **< 2e-16 ***** |
| Age:C:Fluke-infected | -3.18e-03 | 2.37e-01 | 0.989 |
| Age:A:Fluke-infected | -1.26e-01 | 1.44e-01 | 0.381 |
| Age:SA:Fluke-infected | 3.18e-03 | 2.37e-01 | 0.990 |

1. Faith’s PD

|  | Estimate | Std. Error | p-value |
| --- | --- | --- | --- |
| Sex:M | 1.19e-03 | 5.56e-04 | **0.033 *** |
| Area:S-NP / BF-NP | -1.73e-03 | 6.49e-04 | **0.008 **** |
| Area:N-FE / BF-NP | -7.90e-04 | 9.96e-04 | 0.428 |
| Area:N-FE / S-NP | 9.40e-04 | 9.40e-04 | 0.318 |
| Age:C / SA | -1.05e-03 | 7.96e-04 | 0.187 |
| Age:A / SA | -9.20e-05 | 8.50e-04 | 0.914 |
| Age:SA / C | 1.05e-03 | 7.96e-04 | 0.187 |
| Fluke-infected | -3.07e-03 | 1.62e-03 | 0.059 |
| Season:Aug / Jan | 4.10e-04 | 1.10e-03 | 0.709 |
| Season:Sep / Jan | -1.11e-03 | 9.19e-04 | 0.230 |
| Season:Oct / Jan | -1.56e-03 | 8.77e-04 | 0.076 |
| Season:Nov / Jan | -4.31e-03 | 7.94e-04 | **9.82e-08 ***** |
| Season:Dec / Jan | -3.65e-03 | 7.36e-04 | **1.03e-06 ***** |
| Season:Sep / Aug | -1.52e-03 | 1.18e-03 | 0.198 |
| Season:Oct / Aug | -1.97e-03 | 1.14e-03 | 0.085 |
| Season:Nov / Aug | -4.72e-03 | 1.09e-03 | **1.93e-05 ***** |
| Season:Dec / Aug | -4.06e-03 | 1.08e-03 | **0.0002 ***** |
| Season:Oct / Sep | -4.54e-04 | 9.70e-04 | 0.640 |
| Season:Nov / Sep | -3.21e-03 | 8.90e-04 | **0.0004 ***** |
| Season:Dec / Sep | -2.55e-03 | 9.07e-04 | **0.005 **** |
| Season:Nov / Oct | -2.75e-03 | 8.60e-04 | **0.001 **** |
| Season:Dec / Oct | -2.09e-03 | 8.70e-04 | **0.017 *** |
| Season:Dec / Nov | 6.58e-04 | 7.85e-04 | 0.402 |
| Seqdepth | -6.64e-08 | 2.63e-09 | **< 2e-16 ***** |
| Age:C:Fluke-infected | 1.80e-03 | 2.85e-03 | 0.529 |
| Age:A:Fluke-infected | 1.38e-03 | 1.83e-03 | 0.452 |
| Age:SA:Fluke-infected | -1.80e-03 | 2.85e-03 | 0.529 |

**Table S4** Effect of intrinsic and extrinsic factors on three metrics of microbial beta diversity in red deer with a) unweighted and b) weighted UniFrac distances. Permutation test for capscale under reduced model, terms added sequentially, permutation=free and No. permutations= 9999. Significant p-values in bold. Significance codes: *** p < 0.001, ** p < 0.01, * p < 0.05.

a

|  | DF | SumOfSqs | F | Pr(>F) |
| --- | --- | --- | --- | --- |
| Sex | 1 | 0.15 | 1.41 | 0.064 |
| Area | 2 | 1.03 | 4.80 | **0.0001***** |
| Age | 2 | 0.56 | 2.59 | **0.0001***** |
| Fluke prevalence | 1 | 0.16 | 1.52 | **0.038*** |
| HuntingMonth | 5 | 1.12 | 2.10 | **0.0001***** |
| Age: Fluke prevalence | 2 | 0.18 | 0.82 | 0.838 |
| Residual | 403 | 43.09 |  |  |
|  |  |  |  |  |
| Sex | 1 | 0.15 | 1.41 | 0.062 |
| Area | 2 | 1.03 | 4.81 | **0.0001***** |
| Age | 2 | 0.56 | 2.60 | **0.0001***** |
| Fluke infestation intensity | 1 | 0.23 | 2.12 | **0.008**** |
| HuntingMonth | 5 | 1.14 | 2.13 | **0.0001***** |
| Residual | 405 | 43.19 |  |  |

b

|  | DF | SumOfSqs | F | Pr(>F) |
| --- | --- | --- | --- | --- |
| Sex | 1 | 0.02 | 1.58 | 0.088 |
| Area | 2 | 0.47 | 18.95 | **0.0001***** |
| Age | 2 | 0.07 | 2.77 | **0.0003***** |
| Fluke prevalence | 1 | 0.01 | 0.95 | 0.438 |
| HuntingMonth | 5 | 0.15 | 2.45 | **0.0001***** |
| Age: Fluke prevalence | 2 | 0.02 | 0.71 | 0.731 |
| Residual | 403 | 4.95 |  |  |
|  | | | | |
| Sex | 1 | 0.02 | 1.58 | 0.086 |
| Area | 2 | 0.47 | 19.05 | **0.0001***** |
| Age | 2 | 0.07 | 2.79 | **0.0003***** |
| Fluke infestation intensity | 1 | 0.03 | 2.69 | **0.037*** |
| HuntingMonth | 5 | 0.15 | 2.45 | **0.0001***** |
| Residual | 405 | 4.95 |  |  |


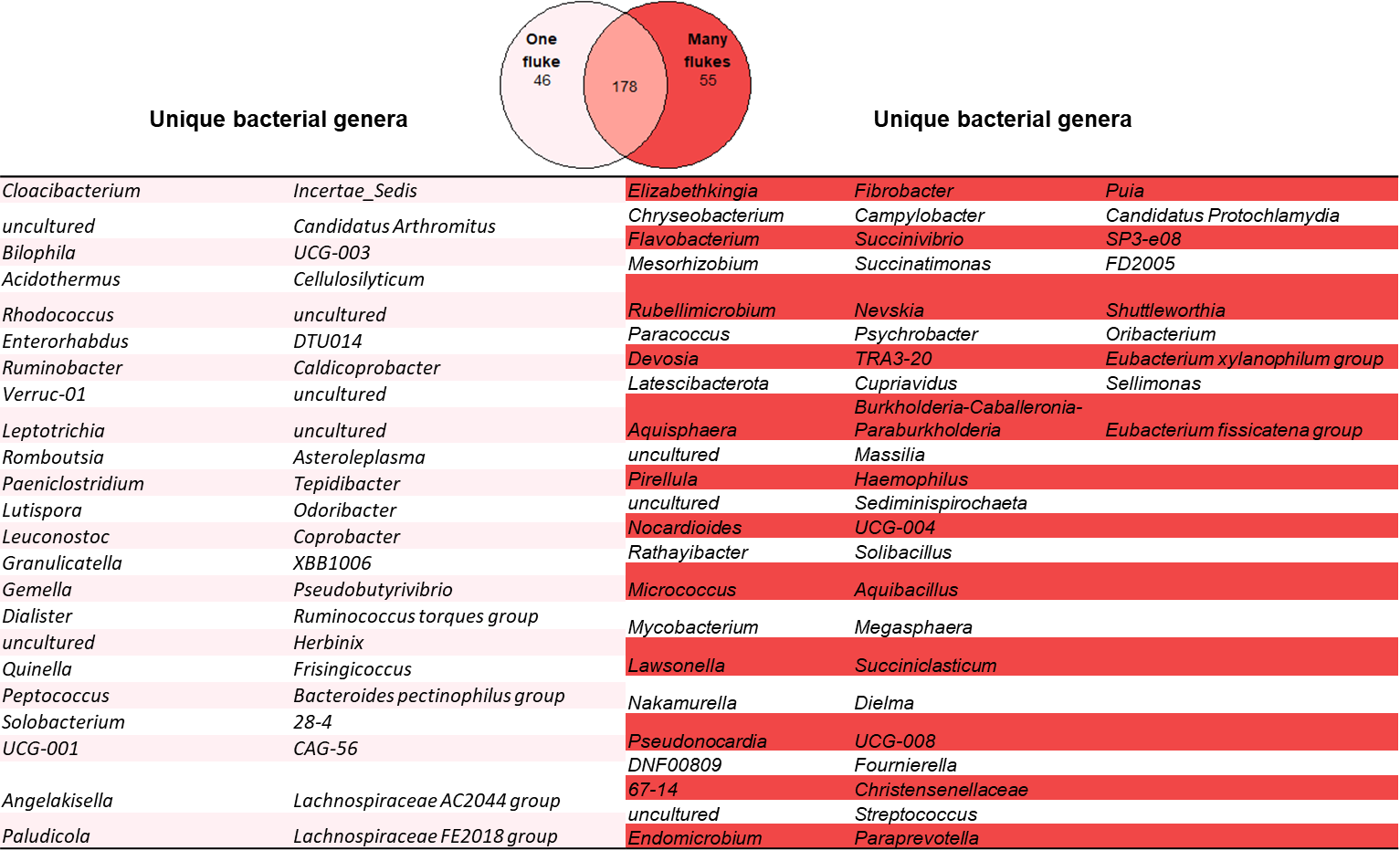
**Table S5** Gut bacterial taxa on genus level that were unique to individuals carrying one or many liver flukes, respectively.
